# Factors associated with access to physical rehabilitation for victims of traffic accidents

**DOI:** 10.1590/S1518-8787.2017051006429

**Published:** 2017-06-13

**Authors:** Kelienny de Meneses Sousa, Wagner Ivan Fonsêca de Oliveira, Emanuel Augusto Alves, Zenewton André da Silva Gama

**Affiliations:** I Programa de Pós-Graduação em Saúde Coletiva. Universidade Federal do Rio Grande do Norte. Natal, RN, Brasil; II Instituto Federal de Educação, Ciência e Tecnologia do Rio Grande do Norte. São Paulo do Potengi, RN, Brasil; III Curso de Medicina. Universidade Federal do Rio Grande do Norte. Natal, RN, Brasil; IV Departamento de Saúde Coletiva. Universidade Federal do Rio Grande do Norte. Natal, RN, Brasil

**Keywords:** Accidents, Traffic, Rehabilitation Services, Health Services Accessibility, Quality of Health Care

## Abstract

**OBJECTIVE:**

Evaluate the level of access to physical rehabilitation for survivors of traffic accidents and the associated factors.

**METHODS:**

A cross-sectional study performed in Natal, Northeastern Brazil, through a telephone survey of 155 victims of traffic accidents admitted to an emergency hospital between January and August of 2013, with a diagnosis of fracture, traumatic brain injury or amputation. Participants were identified in the database of the reference hospital for care of traffic accident victims. We calculated point estimates and confidence interval (95%CI) for the frequency of subjects who had access, in addition to multivariate analysis (logistic regression) between access (dependent variable) and sociodemographic, clinical, and assistance variables.

**RESULTS:**

Among the 155 respondents, the majority were adolescents and adults between 15–29 years of age (47.7%), men (82.6%), education up to high school (92.3%), income of up to two minimum wages (78.0%) and bikers (75.5%). Although 85.8% of traffic accident survivors reported the need for physical rehabilitation, there was little access (51.6%; 95%CI 43.7–59.4) and a delay to start the physical rehabilitation (average = 67 days). We classified factors associated with access to physical rehabilitation as: (i) unmodifiable individuals in the short term – family income greater than two minimum wages (OR = 3.7), informal worker (OR = 0.11) or unemployed (OR = 0.15) and possession of a private health care plan (OR = 0.07); and (ii) assistance modifiable by service management – written referral for physical rehabilitation (OR = 27.5) and perceived need of physical rehabilitation (OR = 10).

**CONCLUSIONS:**

This study found a low and slow access to physical rehabilitation for individuals potentially in need. The associated factors were the organizational processes of health care (health information and referral) and social determinants (income, occupation and private health care plan).

## RESUMO

**OBJETIVO:**

Avaliar o nível de acesso à reabilitação física para sobreviventes de acidentes de trânsito e seus fatores associados.

**MÉTODOS:**

Estudo transversal realizado em Natal, Nordeste do Brasil, com inquérito telefônico a 155 vítimas de acidentes de trânsito atendidos no hospital de emergência, entre janeiro e agosto de 2013, com diagnóstico de fratura, traumatismo cranioencefálico ou amputação. Os participantes foram identificados no banco de dados do hospital referência para atendimentos de acidentes de trânsito. Calculou-se a estimativa pontual e o intervalo de confiança (IC95%) da porcentagem de acesso e tempo para acesso, além de análise multivariada (regressão logística) entre o acesso (variável dependente) e as variáveis sociodemográficas, clínicas e assistenciais.

**RESULTADOS:**

Entre os 155 entrevistados, predominaram jovens e adultos de 15–29 anos (47,7%), sexo masculino (82,6%), escolaridade até o ensino médio (92,3%), renda de até dois salários mínimos (78,0%) e motociclistas (75,5%). Embora 85,8% dos sobreviventes de acidentes de trânsito tivessem relatado a necessidade de reabilitação física, houve baixo acesso geral (51,6%; IC95% 43,7–59,4) e demora para início da reabilitação física (média = 67 dias). Os fatores associados ao acesso à reabilitação física classificaram-se em: (i) individuais não modificáveis em curto prazo – renda familiar maior que dois salários mínimos (OR = 3,7), ser trabalhador informal (OR = 0,11) ou desempregado (OR = 0,15) e ter plano privado de saúde (OR = 0,07); e (ii) assistenciais modificáveis pela gestão do serviço – encaminhamento escrito para reabilitação física (OR = 27,5) e necessidade percebida de reabilitação física (OR = 10).

**CONCLUSÕES:**

Este estudo encontrou um baixo e demorado acesso à reabilitação física para indivíduos potencialmente necessitados. Os fatores associados foram os processos organizativos dos cuidados em saúde (encaminhamento e informação em saúde) e os determinantes sociais (renda, ocupação e plano privado de saúde).

## INTRODUCTION

Traffic accidents (TA), a global public health problem, cause approximately 1,240,000 deaths and 20 to 50 million physical injuries and disabilities every year^29^. On the list of 101 nations with highest mortality rates for this kind of urban violence, Brazil ranks fourth (23 deaths/100,000 inhabitants)^28^. The victims are usually men, young workers, older adults, bikers, and pedestrians^2^ ^,^ ^11^ ^,^ ^28^.

The TA victims require full, urgent, life-saving health care, and rehabilitation for full clinical and functional restoration. The TA survivors usually suffer physical, psychological, and social sequelae. Physically, mobility restrictions can decrease or inhibit completely a person’s functionality and independence and generate incapacity for work^4^. In addition, psychosomatic diseases^12^ and social isolation are individual consequences interrelated with the physical damage that also has a collective impact.

However, many TA survivors face another type of violence: the State’s failure and the health systems’ inefficiency to ensure the right to health, including physical rehabilitation (PR). To deprive the victims of PR services unnecessarily increases their pain and suffering, aggravates the health consequences of the injury^24^, limits social activities and participation^4^, reduces the quality of life, and increases spending on health and social security services^9^. These issues are consistent in the literature and show that access to these services is crucial to ensure equal opportunities and quality of life for survivors.

Easy and timely access is a priority dimension to a high-quality health system^30^ and implies that a risk population can utilize health services in a manner proportional and tailored to their existent needs^1^. Its presence precedes individual care e enables other key dimensions of quality (safety, user-centered care, effectiveness, and efficiency). This way, the worst health system is the one that does not guarantee access to the population in need.

The quality of PR services is still a subject seldom discussed in Public Health, with persistent gaps on access and other dimensions of quality. Some studies have found a certain devaluation of PR by administrators, in addition to inadequate offer and irregular geographical distribution offer^8^ ^,^ ^14^. This is worrisome when we reflect on the importance of PR when fighting the TA pandemic.

Within the Unified Health System (SUS), access to PR for survivors of TA depends on the appropriate transition between the hospital and outpatient rehabilitation care, provided by the Care Network for Urgencies and Emergencies^17^ and in the Care Network for Disabled People^16^. However, the deployment and coordination of these networks are still incipient, damaging the completeness of the care.

Despite the efforts of Brazilian traffic laws to reduce deaths by TA, its occurrence is still high, especially in young people. The city of Natal, State of Rio Grande do Norte, has data consistent with the Brazilian reality in regards to juvenile mortality, which had, in 2012, a significant increase of 98.3% when compared to 2011^28^.

Considering these priors, this study aimed to estimate the level of access to PR by TA victims and the associated factors in a Brazilian capital particularly affected by the problem. The access estimate provides a parameter to examine the system’s capability to serve this population and the associated factors, when modifiable, may represent opportunities for improving the health system’s quality.

## METHODS

Cross-sectional, observational study, carried out in 2014, through a telephone survey of TA survivors admitted in the emergency department of the reference hospital for traumas in the State of Rio Grande do Norte, located in the city of Natal. According to the studied hospital’s database, from January to August 2013, there were 5,367 TA, of which 2,795 involved residents of Natal. The study population included TA victims living in Natal, RN, treated in the studied hospital, diagnosed with fractures, traumatic brain injury or amputation, conditions that justify the need for PR. The total population of the study was 612 subjects.

We calculated the sample size to estimate the proportion of individuals with access to PR to get a precision of 5% with 95% confidence index. After adjusting for finite populations, the number of intended subjects was 235. Random sampling was performed to ensure representativeness of the sample in relation to the population of study. Subjects with phone contacts unavailable or nonexistent after four attempts were randomly replaced to complete the desired sample size.

Data collection took place between March and July 2014. Individuals and their phone contacts were identified through the database provided by the hospital. Patients who had been released or their guardians were located by telephone calls so two researchers could apply the questionnaire. The average duration of each telephone interview was 13 minutes and 30 seconds.

Researchers developed the instrument used after previous qualitative study, which identified potential barriers to PR access^23^. It contained 25 objective questions about demographic, accident related, clinical, and assistance aspects. The instrument was tested in a pilot study with part of the desired sample (8.9%; 21 subjects) in order to ascertain its feasibility, relevance, and appropriateness of the terminology.

From the questionnaire we collected the following variables of interest: (1) dependent: general access to PR, which included access to private or public service, and public access to PR (yes, no); (2) independent: sociodemographic (gender, age, education, family income, marital status, private health care plan, and occupation), traffic accident-related (type of victim, transportation, and accident), clinical (self-care, ambulation, mobility, type and location of the injury, perceived need of PR) and assistance (clinical treatment, in-patient duration, hospital rehabilitation, information on PR, PR referral).

The information collected was analyzed by the statistical software SPSS, version 22.0. After descriptive statistics, we calculated point estimates and intervals (95%) for the frequency of subjects who had access (public or otherwise) and the average time for starting the rehabilitation.

Associations between categorical variables were quantified with Pearson’s Chi-squared test. The variables associated with access with p < 0.20 in bivariate analysis were included in the logistic regression models, stepwise, with p < 0.05 and 95%CI. We developed two logistic regression models: “general access to PR” and “public access to PR”. As the outcomes are binary, the associations were measured by odds ratio (OR). The quality of the regression models’ adjustment was evaluated by the Hosmer-Lemeshow test and Nagelkerke’s R-squared.

This study was approved by the Research Ethics Committee of the Hospital Universitário Onofre Lopes of the Universidade Federal do Rio Grande do Norte (Protocol 611.492/2014).

## RESULTS

Six-hundred and twelve subjects met the inclusion criteria. The estimated sample size was 235 individuals. Even with the expansion of telephone calls for the whole study population (n = 612), 155 subjects were interviewed (66% response percentage). There was only one refusal. Detailed data on the characteristics of the sample are shown in Table 1.

**Table 1:** Sample characterization (n = 155) concerning socio-demographic, accident, functional clinical and assistance variables. Natal, State of Rio Grande do Norte, Brazil, 2014.

Variable	Frequency	Percentage
Sociodemographic variables		

Gender		
Male	128	82.6
Female	27	17.4
Age (years)		
7–14	10	6.4
15–29	74	47.7
30–65	59	38.0
66–80	12	7.7
Marital status		
Married	54	34.8
Single	101	65.1
Education		
Middle school	70	45.2
High school	73	47.1
Higher education	12	7.7
Occupation		
Student	23	14.8
Retiree	14	9.0
Unemployed	12	7.7
Informal worker	29	18.7
Employed	77	49.7
Income (minimum wage)		
1–2	121	78.0
> 2	34	21.9
Private health care plan		
Yes	13	8.4
No	142	91.6

Traffic accident variables		

Type of accident		
Fall	17	11.0
Run over	21	13.5
Collision	117	75.4
Type of vehicle		
Motorcycle	114	73.5
Automobile	11	7.1
Non-motorized transport	30	19.3
Type of victim		
Driver	106	68.3
Passenger	27	17.4
Pedestrian	22	14.1

Clinical functional variables		

Lesion diagnosis		
Amputation		
Yes	7	4.5
No	148	95.9
Fracture		
Yes	129	83.2
No	26	16.8
Traumatic brain injury		
Yes	18	11.6
No	137	88.3
Soft tissue injury		
Yes	15	9.3
No	140	90.3
Site of injury		
Upper limbs	72	46.5
Lower limbs	89	57.4
Functional limitation		
Self-care	131	84.5
Ambulation	109	70.3
Mobility	141	91.0
Difficulty of return to work	101	95.2
Perceived need for PR		
Yes	133	85.8
No	22	14.2
Time until return to work (months)		
0–2	21	26.9
3–4	20	25.6
6–14	37	47.4
Loss or inability of employment*	27	25.4

Clinical assistance variables		

Orthopedic surgery		
Yes	108	69.7
No	46	30.3
Immobilization with plaster cast		
Yes	45	29.0
No	110	71.0
Hospital rehabilitation		
Yes	27	17.4
No	128	82.6
Length of stay (days)		
0–2	39	25.2
3–8	41	26.5
9–22	37	23.9
23–120	38	24.5
Referral to PR		
Yes	80	51.6
No	75	48.4
Information about PR		
Yes	86	55.5
No	69	44.5

PR: physical rehabilitation; TA: traffic accidents

* In the analysis of work-related variables we included only individuals who were working at the time of the TA (n = 106), no t equivalent to the total respondents (n = 155).

Most frequent among the victims were young people from 15 to 29 years of age, men, single, education up to high school, income of up to two minimum wages and economically active. Almost all respondents depended solely on the public health system (Table 1).

The most frequent accidents were with motorcycles and the recurring injury was a fracture, resulting in limitations in self-care and ambulation activities. In accordance with the criteria for inclusion in the sample, which were based on the potential need for PR, most respondents reported they needed PR (Table 1).

Regarding health assistance, most needed orthopedic surgery. Surgical fixation was the most appropriate intervention (70.9% of cases). Although 75.6% of respondents remained in the hospital for a period of up to 22 days, only 17.4% started rehabilitation in the hospital. Slightly over half (51.6%) of patients received referrals for PR when they were released from the hospital, and a slightly larger number (55.5%) received orientation from hospital staff about PR (Table 1).

We also identified occupational damage, because 1/4 of workers lost their jobs or became incapacitated to work. Among those who exerted any labor activity (n = 106), the largest portion of the sample, almost all (95.2%) mentioned difficulty in returning to work. The average time to return to work was 146 days (95%CI 134–157) (Table 1).

In regards to general access to PR services, approximately half of the respondents (51.6%; 95%CI 43.7–59.4) managed to have access to them, of which 32.9% accessed it through public services and 18.7% through private services. The time for that access was 74 days in public service and 56 days in private (Table 2).

**Tabela 2:** Estimativa do acesso e tempo de acesso à reabilitação física para vítimas de acidentes de trânsito. Natal, RN, 2014.

Variable	Average (n)	Frequency (n)	Percentage	95CI%
Access (yes)				
General	-	80 (155)	51.6	43.7–59.4
Public	-	51 (155)	32.9	25.5–40.2
Private	-	29 (155)	18.7	12.6–24.7

Time of access (in days)				
General	66.6 (80)	-	-	51.0–82.3
Public	74.0 (51)	-	-	62.1–85.8
Private	56.4 (29)	-	-	49.7–63.0

Numbers of cases presented in parentheses.

The bivariate analysis identified 11 variables associated with access (p < 0.05); three related to the social condition, one related to the type of accident, three related to clinical condition and five related to health assistance (Table 3).

**Table 3:** Estimate of general and public access to PR for each variable of the bivariate analysis. Natal, State of Rio Grande do Norte, Brazil, 2014. (n = 155)

Variable	General access (%)	OR	95CI%	p	Public access (%)	OR	95CI%	p
Income (minimum wage)a								
> 2	72.7	3.2	1.37–7.45	0.005	42.4	1.7	0.78–3.84	0.168
1-2	45.5				29.8			
Education								
Higher education^b^	75.0	-	-	0.177	33.3	-	-	0.939
High school	53.4	0.28	0.07–1.12	0.073	31.5	1.04	0.28–3.82	0.949
Middle school	45.7	0.38	0.10–1.52	0.174	34.3	0.92	0.25–3.36	0.900
Marital status								
Married	58.5	0.65	0.33–1.28	0.217	41.5	0.54	0.27–1.10	0.090
Single	48.0				28.0			
Occupation^a^								
Employed^b^	63.6	-	-	0.009	39.0	-	-	0.076
Retiree	64.3	0.44	0.17–1.13	0.089	50.0	0.68	0.25–1.86	0.460
Unemployed	33.3	1.02	0.31–3.37	0.963	33.3	1.56	0.45–4.91	0.442
Informal worker	27.6	0.28	0.08–1.03	0.056	10.3	0.78	0.21–2.83	0.709
Student	43.5	0.21	0.08–0.55	0.001	30.4	0.18	0.05–0.65	0.009
Private health plan^a^								
Yes	61.5	1.56	0.48–4.98	0.454	7.70	0.17	0.02–1.21	0.043
No	50.7				35.2			
Type of victim								
Pedestrian^b^	38.1	-	-	0.198	14.3	-	-	0.187
Passenger	42.3	2.07	0.79–5.41	0.136	34.6	3.30	0.91 – 11.9	0.068
Driver	56.1	1.19	0.36–3.86	0.770	35.5	3.17	0.73 – 13.7	0.122
Type of accident^a^								
Collision^b^	55.2	-	-	0.227	37.9	-	-	0.051
Run over	42.9	0.44	0.15–1.28	0.132	14.3	0.35	0.09–0.28	0.115
Fall	35.3	0.61	0.23–1.56	0.301	17.6	0.27	0.07–0.98	0.046
Motorcycle								
Yes	58.3	1.44	0.69–3.00	0.329	35.9	1.80	0.78–4.17	0.164
No	44.7				23.7			
Lower limbs injury								
Yes	56.2	1.53	0.81–2.91	0.186	33.7	1.09	0.55–2.14	0.804
No	45.5				31.8			
Self-care limitations								
Yes	55.0	2.44	0.97–6.09	0.051	33.6	1.22	0.47–3.18	0.672
No	33.3				29.2			
Perceived need for PR^a^								
Yes	58.6	14.2	3.18–63.1	0.000	37.6	12.6	1.65–96.9	0.002
No	9.1				4.50			
Orthopedic amputation^a^								
Yes	85.7	6.0	0.70–51.1	0.065	71.4	5.54	1.03–29.6	0.026
No	50.0				31.1			
Soft tissue injury^a^								
Yes	40.0	0.59	0.20–1.76	0.489	6.70	0.12	0.01–1.00	0.023
No	52.9				37.7			
Written referral^a^								
Yes	82.5	20.5	9.06–46.5	0.000	57.5	18.9	6.9–51.2	0.000
No	18.7				6.7			
Orthopedic surgery^a^								
Yes	63.9	5.79	2.65–12.6	0.000	39.8	3.22	1.37–7.56	0.006
No	23.4				17.0			
Hospital rehabilitation								
Yes	63.0	1.75	0.74–4.12	0.194	44.4	1.82	0.78–4.26	0.160
No	49.2				30.5			
Immobilization with plaster cast^a^								
Yes	58.2	0.39	0.19–0.81	0.011	38.2	0.41	0.18–0.95	0.029
No	35.6				20.0			
Information about PR^a^								
Yes	74.4	9.63	4.6–20.20	0.000	48.8	6.34	2.8–14.42	0.000
No	23.2				13.0			
Length of stay (days)^a^								
0–2^b^	23.1	-	-	0.001	10.3	-	-	0.014
3–8	65.9	6.42	2.40–17.2	0.000	39.0	5.60	1.67–18.7	0.005
9–22	56.8	4.37	1.62–11.7	0.003	37.8	5.32	1.56–18.2	0.008
23–120	60.5	5.11	1.90–13.7	0.001	44.7	7.08	2.10–23.9	0.002

PR: physical rehabilitation

^a^ Variable with p < 0.05.

^b^ Category-reference.

The general access to PR regression model showed a greater chance of access for individuals with income greater than two minimum wages (MW) compared to those with income of up to two MW (OR = 3.7). The chances of getting rehabilitation treatment were also higher for individuals with perceived need of PR (OR = 10.0) and referral for PR (OR = 27.5). However, unemployed individuals (OR = 0.15) or informal workers (OR = 0.11) showed a reduced chance of access to PR in relation to those employed. This model’s adjustment quality had a statistical significance of 0.981 on the Hosmer-Lemeshow test and explains 62.1% of the access variability (Table 4).

**Table 4:** Logistic regression models of factors associated with general and public access to physical rehabilitation. Natal, State of Rio Grande do Norte, Brazil, 2014. (n = 155)

Variable	Categories	n (% access)	Adj OR	95CI%	p
Model 1 – General access to physical rehabilitation^a^					

Occupation	Employed^c^	49 (63.6)			0.018
	Unemployed	4 (33.3)	0.15	0.02–0.96	0.046
	Informal worker	8 (27.6)	0.11	0.03–0.43	0.001
Family income (minimum wage)	> 2	24 (72.7)	3.72	1.07–13.00	0.039
	1-2	55(45.5)			
Perceived need for PR	Yes	78 (58.6)	10.00	1,30–76.53	0.027
	No	2 (9.1)			
Written referral	Yes	66 (82.5)	27.50	9.52–79.42	< 0.001
	No	14 (18.7)			

Model 2 – General access to physical rehabilitation in the public health system^b^					

Private health care plan	Yes	1 (7.7)	0.07	0.008–0.59	0.014
	No	50 (35.2)			
Written referral	Yes	46 (57.5)	23.00	8.22–68.24	< 0.001
	No	5 (6.7)			

PR: physical rehabilitation

^a^ Hosmer–Lemeshow test: p = 0.98; Nagelkerke’s R squared: p = 0.621.

^b^ Hosmer–Lemeshow test: p = 0.989; Nagelkerke’s R squared: p = 0.451.

^c^ Category-reference.

Numbers of cases presented in parentheses.

As for public access to PR, the model presented referral with a strong positive association (OR = 23.0) and a private health care plan with a negative association (OR = 0.07). This model also showed good adjustment quality in the Hosmer-Lemeshow test, with a statistical significance of 0.989, and the proportion of variability explained by the model was 45.1% (Table 4).

## DISCUSSION

This study contributes to understanding PR accessibility for victims of traffic accidents in a city in Northeastern Brazil. Due to the scarcity of studies in this area, it presents original information that indicates access problems and factors associated with an inefficient health system to care for the victims of this global epidemic. It also indicates modifiable factors that can improve the quality of care through social interventions and reorientation of health actions by public administrators.

The profile of the TA victim in this study (young adults, bikers, economically active, of low income and low education) is consistent with the national scenario and confirms their social vulnerability^2^ ^,^ ^11^ ^,^ ^28^. Even with the incentive of traffic laws, the morbidity and mortality in this vulnerable population profile continue to be high^2^ ^,^ ^11^, because the loss of functional capacity and social participation of this population exacerbates existent social inequalities^15^.

Access levels were low because only half of those who needed PR managed to obtain the service. We did not find studies with access estimated for this population, but only studies about access in other areas (women’s health and elderly health)^18^ ^,^ ^21^ or in a distinct specialty (cardiac rehabilitation)^22^. For example, in patients who suffered a stroke, we found a higher percentage of access to rehabilitation (67.1%) in a cross-sectional study performed in João Pessoa, State of Paraiba^21^ and even higher (90%) in a cohort study conducted in the United States^20^. This shows that these data can vary in different health systems.

The results indicate the supporting role of PR in SUS services, something that contradicts its principle of care integrality. Only one out of every three patients had access through the public system, a result always lower than those identified for other health services: 50.9% for care in urban areas, in a health center^13^; 71.5% for access to exams^18^; and 93.5% for care at health centers in São Paulo, State of São Paulo^3^.

Another problem is the delay to start the rehabilitation. For stroke patients and children with cerebral palsy, the access time varied from one to six months^21^ ^,^ ^25^, which shows that the long wait is usually an instituted aspect of PR. A delay in the start of PR, whether in public or private service, hurts the concept of access based on the timely offer of services and conditions appropriate to affect the health outcomes positively^30^. Besides generating user dissatisfaction, the delay to start rehabilitation can resonate in other dimensions of quality, as in clinical effectiveness^24^ and patient safety, because delay complications are unnecessary damages associated with the service that harms health outcomes and increases costs^30^.

The difficulty of access can be a consequence of an incomplete model of care that does not cover all the needs of the population subject to TA (health promotion and protection, prevention of TA, urgent care and rehabilitation of injuries). Initiatives such as the Care Network for Urgencies and Emergencies^17^ and the Care Network for Disabled People^16^ are partial components of a comprehensive care model; however, the deployment and coordination of these policies are still fledgling in the context of this study. The consequences of no access to PR are many, from unacceptable prolonging of these victims’ pain and suffering to the generation of unemployment and family and social instability, situations that are part of the perpetuation of violence after the TA and the insufficient protection of these individuals by the Brazilian State.

We classified factors associated with access to PR as individual non-modifiable in the short-term (family income, occupation, private health care plan) and assistance modifiable by service management (written referral and perceived need) (Figure).

**Figure:**
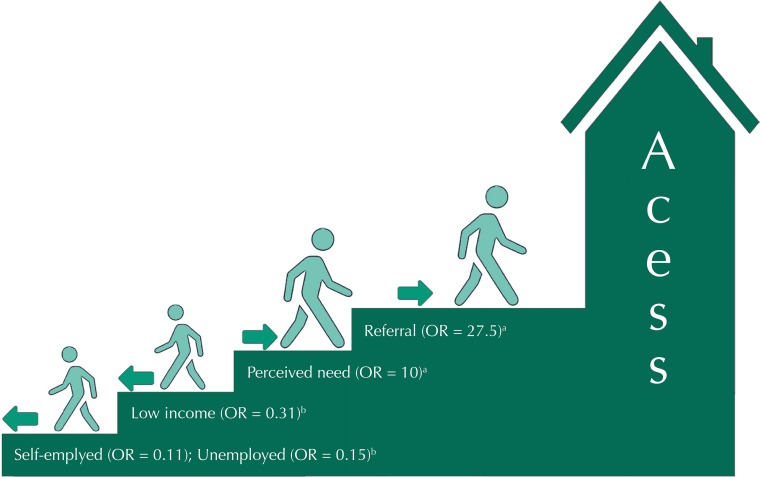
Analysis model of access to physical rehabilitation determinants. Natal, State of Rio Grande do Norte, Brazil, 2014.

^a^ Organizational factors modifiable by service management.

^b^ Individual factors nonmodifiable in the short term.

Individual aspects were termed “not modifiable in the short term” because they are particular difficulties of users, and their solutions involve a higher level of complexity. Consistent with population studies^3^ ^,^ ^15^ ^,^ ^26^, low income was related to a lower use of health services. Another issue are unemployed individuals and informal workers, who have the greater difficulty of access, probably because of the precariousness of labor rights for their own health care. This negative influence of occupational activity on access has also been found at a study by Travassos et al.^26^ This reveals that the gratuity of the services is not enough, but we need to consider the particular limitations of the victims, such as financial expenses and time spent away from labor activities or for transportation to the treatment locale.

Private health care plan was unrelated to general access. This result contrasts to the literature because a private plan usually relates to greater access to health services^3^ ^,^ ^6^ ^,^ ^13^ ^,^ ^26^, this may be a local reality or of that specific population. However, having a private plan was significantly associated with lower access to public service, an expected result since the patients are able to choose.

Although some studies have found a reduction of inequities in access to the Brazilian health system^19^ ^,^ ^27^, our results suggest that this scenario is still significant in the capital of study, especially concerning rehabilitation. Modifying individual factors such as those mentioned above involves interventions on social issues based on social determinants of health, complex actions that require intersectoral efforts and different public administration plans^5^ ^,^ ^29^.

Of the modifiable aspects, the self-perception of rehabilitation need increases the likelihood of access. This confirms national studies^3^ ^,^ ^13^ ^,^ ^18^, in which the perceived need influences the demand and use of the services. A study involving individuals with disabilities in China found that, among those who expressed any need of rehabilitation (75%), only 27% obtained access to them^31^. In our study, 85.8% of participants reported that need and 58.6% of those got the treatment they sought.

Despite the perceived necessity being a feature of the user, in this study, it was considered as an aspect of services by their close relationship with the information offered by health professionals. Informing the user is an ethical precept of every health worker and facilitates proper and responsive movement of users at various levels of the system, turning them into regulators of access themselves^7^. The service management should standardize the provision of information about PR through a user-centered and humanized care, in addition to monitoring the adherence of professionals to such standards.

The medical referral was the main determinant of access, and it is a modifiable factor by service management. It is the historic problem of reference and counterreference^7^ ^,^ ^10^. In this regard, we found that the professionals, although entered into a health system, do not perceive themselves to be in an actual “system”, but isolated from health care networks. In order to ensure access and full assistance to TA victims, it is necessary to amplify the point of view of administrators and professional for care coordination planning between the different points of care in the network (primary care, hospitals, emergency, and rehabilitation).

This study has some limitations. Although it is useful to identify insufficient access, the margin of error of the access estimate is relatively large because the sample size was not ideal at the start, even with the expansion of the telephone calls to the entire study population. It is also possible that there has been some memory bias regarding access time, although in the sample most respondents were young adults interviewed not long after the TA. Additionally, the collection by telephone survey made the research possible research but limited the number of variables collected, and future studies with complementary data collection methods are necessary. As to the inclusion of only subjects with phone contacts, this procedure may have overestimated the access, since the phone can be a social indicator. Still, in light of the social and economic diversities between Brazilian capitals and regions, the extrapolation of the results becomes limited.

Further research is necessary to understand the quality of PR services relative to other dimensions of quality in addition to access, such as effectiveness, patient safety, fairness, efficiency and user-centered care. Furthermore, we suggest the development of intervention studies about the factors associated with access to PR identified in this study, especially those considered modifiable. Studies about clinical and economic outcomes related to the quality of the PR are also necessary.

In conclusion, the access to physical rehabilitation services was insufficient to meet the needs of traffic accident victims. The factors that must be modified to improve access include complex situations and other of a possible resolution with an effective management of care quality. Therefore, the struggle to achieve integrality of health care, which includes easy access to rehabilitation, transcends the field of awareness and involvement of stakeholders (users, professionals, and health administrators). It is necessary to exploit situational analysis based on access estimates and associated factors for a rational, effective and feasible planning of interventions and care models for victims of traffic accidents.
